# Progress and Challenges in the Search for the Mechanisms of Pulsatile Gonadotropin-Releasing Hormone Secretion

**DOI:** 10.3389/fendo.2017.00180

**Published:** 2017-07-24

**Authors:** Stephanie Constantin

**Affiliations:** ^1^Cellular and Developmental Neurobiology Section, National Institute of Neurological Disorders and Stroke, National Institutes of Health, Bethesda, MD, United States

**Keywords:** gonadotropin-releasing hormone release, preovulatory surge, gonadotropin-releasing hormone pulsatility, kisspeptin, electrophysiology, calcium imaging

## Abstract

Fertility relies on the proper functioning of the hypothalamic–pituitary–gonadal axis. The hormonal cascade begins with hypothalamic neurons secreting gonadotropin-releasing hormone (GnRH) into the hypophyseal portal system. In turn, the GnRH-activated gonadotrophs in the anterior pituitary release gonadotropins, which then act on the gonads to regulate gametogenesis and sex steroidogenesis. Finally, sex steroids close this axis by feeding back to the hypothalamus. Despite this seeming straightforwardness, the axis is orchestrated by a complex neuronal network in the central nervous system. For reproductive success, GnRH neurons, the final output of this network, must integrate and translate a wide range of cues, both environmental and physiological, to the gonadotrophs *via* pulsatile GnRH secretion. This secretory profile is critical for gonadotropic function, yet the mechanisms underlying these pulses remain unknown. Literature supports both intrinsically and extrinsically driven GnRH neuronal activity. However, the caveat of the techniques supporting either one of the two hypotheses is the gap between events recorded at a single-cell level and GnRH secretion measured at the population level. This review aims to compile data about GnRH neuronal activity focusing on the physiological output, GnRH secretion.

## Introduction

Fertility and its onset, puberty, are integrated phenomena. A complex network in the central nervous system (CNS), conveying physiological and environmental signals, converges onto neurons secreting gonadotropin-releasing hormone (GnRH). GnRH leads the hormonal cascade, driving gonadotrophs to secrete gonadotropins, which in turn control the gonads, i.e., steroidogenesis and gametogenesis in both sexes and ovulation in females. Thus, GnRH neurons are the output of the CNS for fertility, integrating and encoding cues into a signal readable by gonadotrophs, GnRH. However, GnRH neurons are not an on/off switch but a precise rheostat. GnRH secretion is pulsatile, with changes in amplitude and frequency over time. Yet, the mechanisms by which GnRH neurons generate pulses are unknown. This review summarizes recent data about GnRH neurons with a focus on secretion and the difficulty of answering this fundamental question.

Undoubtedly, cells upstream of GnRH neurons contribute to fertility by helping to provide the homeostatic conditions necessary for survival. However, in this review, the word *fertility* simply refers to the ability to generate gametes and offspring in optimal breeding conditions.

## Neuroanatomical Distribution of GnRH Cell Bodies

Gonadotropin-releasing hormone neurons derive from the olfactory placodes (1, 2) and migrate into the hypothalamus during prenatal development [reviewed in Ref. (3, 4)]. GnRH processes then extend toward the median eminence (ME) (5). This embryonic feature shapes the GnRH neuronal distribution (6). In mouse and rat, the distribution, centered around the preoptic area (POA) and the *organum vasculosum laminae terminalis* (OVLT), respectively (7, 8), is largely confined to the rostral forebrain. In monkey, it expands caudally to the mediobasal hypothalamus (MBH) (9, 10). However, data suggest that the location of the cell bodies is not important to trigger luteinizing hormone (LH) and promote fertility, as long as GnRH nerve terminals reach the hypophyseal portal system. In hypogonadal mice bearing a deletion in the *GnRH* gene (11), transplantation of fetal POA in the rostral third ventricle restores spermatogenesis (12) and pregnancies (13). Similarly, in female monkeys with lesioned MBH, menstrual cycles are restored with transplantation of olfactory placodes in the third ventricle (14). Notably, the pregnancies in mice receiving transplants are initiated by reflex, not spontaneous, ovulation (15), but still indicate gametogenesis and an ovulatory surge occur (16). Two possibilities, extrinsic to GnRH neurons, might explain the absence of spontaneous ovulation in transplanted mice: the required inputs (1) cannot reach transplanted GnRH neurons in their abnormal location and/or (2) are reduced/absent in hypogonadal mice (17). In contrast, in female monkeys, cyclicity was recovered since the inputs were present, i.e., the hypothalamic–pituitary–gonadal (HPG) axis was functional before its disruption. The next section addresses the distinct mechanisms for GnRH secretion leading to ovulation and gametogenesis.

## GnRH Secretion and Fertility

Gonadotropin-releasing hormone neurons have two modes of secretion: surge triggering ovulation, restricted to females, and pulses regulating gametogenesis and sex steroidogenesis, in both sexes. In rat, 90% of GnRH neurons project outside the blood–brain barrier as indicated by Fluorogold retrograde labeling (18). In mouse, only 64% of GnRH neurons are labeled in intact animals but hormonal manipulation labels 88% (19). Unfortunately, peripheral injection of Fluorogold does not discriminate the uptake site. In addition to the ME (20), GnRH neurons exhibit branched processes beyond the blood–brain barrier into the OVLT (21). Thus, the hypophysiotropic proportion of the GnRH population is unknown. Lectin wheat germ agglutinin applied onto the ME reveals an uptake in up to 59% of GnRH neurons (22). While the majority of GnRH neurons probably connect to the ME, a specific number might be irrelevant since few GnRH neurons are needed to acquire and maintain fertility (12, 13, 23). Some GnRH neurons may project to other brain areas, in addition to or instead of the ME and OVLT, and may control additional functions (24, 25).

### Puberty

Puberty is the developmental time an organism acquires its reproductive capacity. Physiologically, puberty coincides with activation of the HPG axis [reviewed in Ref. (26, 27)]. Although this review is not about puberty, I introduce kisspeptin-expressing neurons here (28–30), since puberty onset requires direct contacts onto GnRH neurons, *via* kisspeptin receptor (GPR54) (31).

Kisspeptin neurons are localized in two hypothalamic areas: rostral periventricular area of the third ventricle (RP3V) and the arcuate nucleus (ARC). Both subpopulations express the estrogen receptor alpha and the expression of *Kiss1* gene is sensitive to circulating sex steroids (32, 33). GnRH neurons do not express estrogen receptor alpha (34, 35) and cannot directly integrate gonadal steroid feedback (36). Hence, the role of kisspeptin neurons goes beyond puberty, contributing to fertility throughout life (37). Estradiol has opposite effects on *kiss1* gene expression in the RP3V and ARC in rodents (32, 33). This divergence serves the two GnRH secretory modes. Although the anatomical and functional segregation of the two kisspeptin subpopulations is not obvious in other species (38), rodents help decipher the mechanisms for surge and pulses.

### Preovulatory GnRH Surge

The neurobiology of the preovulatory GnRH surge is reviewed in detail (39, 40). Only a subset of GnRH neurons generates the abrupt release of GnRH into the hypophyseal portal system. In rodents, activated GnRH neurons are immunocytochemically identified by immediate early genes (41, 42). In rat and mouse, ~40% of GnRH neurons, express cFos at the time of the surge (41, 43). Although the OVLT area contains most of the cFos-expressing GnRH neurons, they are found anywhere on the continuum caudal to the OVLT (41, 43). cFos-labeled GnRH neurons exhibit higher spine density (44), indicating increased inputs at the time of the surge. Furthermore, GnRH neurons display entwined dendrites with shared synapses (45), revealing common inputs, despite scattered cell bodies.

Although the surge is not regulated by a single neuronal population (40) and involves cells at the ME (46), kisspeptin is a powerful stimulator of GnRH neurons (47, 48) and direct inputs to GnRH neurons, *via* GPR54, is necessary (31). The RP3V neuronal subpopulation, larger in females and upregulated by estradiol (32), plays a critical role in the activation of GnRH neurons involved in the surge (49). Notably, although physiologically the preovulatory surge is only observed in females, it is not an intrinsic ability of female GnRH neurons but the consequence of female-specific inputs to GnRH neurons. RP3V kisspeptin neurons undergo sex-specific neonatal (50) and prepubertal (51) development, orchestrated by testosterone and estradiol, respectively. Hormonal perturbations altering the sexual dimorphism of RP3V result in LH surges in males and loss of LH surge in females (52).

### GnRH Pulsatility

Gonadectomy releases the HPG axis from sex steroid negative feedback and reveals regular GnRH pulses (53–56). Pulsatility is a critical feature of GnRH secretion and is required for LH secretion (57, 58), underlying LH pulses (59). To date, GnRH pulsatility is still a confounding fact: how do scattered GnRH neurons synchronize to generate pulses? Two possibilities for a pulse generator exist: intrinsic, i.e., GnRH neurons generate pulses on their own or extrinsic, i.e., GnRH neurons are driven by other cell type(s). In the first scenario, synchronization requires connectivity between GnRH neurons, direct or indirect. In the second scenario, synchronization requires connections from a pulse generator to GnRH neurons.

#### Intrinsic Pulse Generator

The hypothesis of an intrinsic pulse generator comes from *in vitro* models for GnRH neurons: (1) mouse cell lines obtained by immortalization, GT1 (60), and (2) primary GnRH cells maintained in organotypic cultures of olfactory placodes, i.e., nasal explants (61–64). Without CNS inputs, these models exhibit pulsatile release of GnRH [GT1 (65–67); nasal explants (64, 68–70)]. One caveat is that nasal explants contain GABAergic and glutamatergic neurons that influence GnRH neuronal activity (71, 72). In both models, GnRH neurons exhibit action potentials (APs) (71, 73, 74) and fluctuations of intracellular calcium concentration ([Ca^2+^]_i_) (75–77), concomitant with bursts of APs (78). GnRH neurons in nasal explants also exhibit synchronized [Ca^2+^]_i_ oscillations (76, 77), supporting connectivity between GnRH neurons.

In immortalized cell lines, gap junctions mediate electrical coupling between GnRH neurons (79–81). Both [Ca^2+^]_i_ waves across GT1 cells (82) and pulsatile GnRH release (80) are gap junction dependent. However, this mechanism might be an adaptation of GT1 cells (83) since *in vivo* data reject coupling between GnRH neurons (84, 85). However, gap junctions between GnRH neurons (84) and surrounding cells (86) could allow signal propagation from one GnRH neuron to another and contribute to the synchronicity. In nasal explants, non-neuronal cells exhibit [Ca^2+^]_i_ oscillations (87) and blocking gap junctions impairs GnRH secretion (86). Hypothetically, if GnRH neurons were electrically connected *in vivo*, electrical activation of a subpopulation of GnRH neurons should propagate through the entire population and evoke an all-or-none GnRH/LH secretory response. However, a linear relationship exists between the number of optogenetically activated GnRH neurons and amplitude of LH pulse, refuting the hypothesis (88).

The alternative to electrical coupling is chemical coupling. GT1 cells on two coverslips within the same chamber exhibit a GnRH secretion profile identical to that of single coverslips, suggesting synchronization through diffusible molecules (66), such as adenosine triphosphate (ATP) or nitric oxide (NO). *In vivo* GnRH neurons express P2X purinoreceptors (89, 90). In nasal explants, ATP contributes to synchronization of GnRH neurons *via* P2X receptors (91), but not basal GnRH neuronal activity (72). In agreement, ATP facilitates, but does not evoke, GnRH release from isolated MBH (92). No physiological data support or refute the role of ATP. While NO contributes to pulsatile secretion at the ME *ex vivo* (93, 94), NO is released at the time of the surge *in vivo* (95, 96). Notably, GnRH neurons do not express NO synthase (NOS) (97), but NO might contribute to the synchronicity of GnRH neurons by modulating their firing in the POA (98). Both NO actions in the ME, *via* endothelial NOS (99, 100), and in the POA, *via* neuronal NOS (98, 101, 102), provide examples of cooperative microenvironments. However, GnRH neurons do not initiate the signal and the second possibility of other cell type(s) driving GnRH neurons dominates.

#### Extrinsic Pulse Generator

*In vitro* ARC–ME fragments exhibit pulsatile release of GnRH (103) and *in vivo* data support the role of the ARC in GnRH pulsatility (104). Increases in the frequency of multiunit activity (MUA) in the ARC are concomitant with LH pulses (105–107). The nature of the cells generating MUA volleys is unknown, but GnRH neurons or GnRH *en passant* fibers are not responsible for them. Estradiol-triggered GnRH surges (107) or kisspeptin-evoked GnRH secretion (108) do not trigger MUA volleys.

Mentioned earlier, the ARC kisspeptin subpopulation is proposed as a pulse generator [reviewed in Ref. (109)]. This subpopulation is not sexually dimorphic and is downregulated by sex steroids (32, 33). These kisspeptin neurons are the central players of an autoregenerative pulsing model. The two peptides they co-express, neurokinin B and dynorphin, are autocrine modulators providing on-/off-switches (110–112). Unfortunately, the model seems incomplete: (1) ARC kisspeptin neurons unequivocally provide an on-switch for GnRH neurons (113), but not an off-switch; kisspeptin evokes long-lasting electrical and calcium responses in GnRH neurons (47, 48, 114, 115), yet only a short activation produces a LH surge (88), (2) neurokinin B evokes GnRH secretion in kisspeptin knockout mice (116), and (3) neurokinin B is present in kisspeptin neurons in humans but dynorphin is not, thus the pulse generator might be species dependent (117). The model might be more convoluted since ARC and RP3V kisspeptin neurons are interconnected (118), co-express glutamate or GABA, respectively (119) and ARC kisspeptin neurons activate GnRH neurons by stimulating RP3V kisspeptin neurons *via* glutamatergic release (112). Thus, the mystery of GnRH pulses remains.

## From GnRH Neuronal Activity to GnRH Secretion

Elucidating how pulsatile GnRH secretion occurs is the key to understanding reproductive neuroendocrinology. However, measuring GnRH secretion is difficult. The GnRH neuronal population is small and a subset generates a pulse, therefore the amount of released GnRH is near threshold detection, even with sensitive radioimmunoassay (70). In addition, access to the hypophyseal portal system requires complex surgery and apparatus (53, 120–122), incompatible with the mouse. Finally, a half-life of GnRH is only 2–4 min. Thus, LH secretion, amplifying and diffusing the GnRH signal to the systemic circulation, is commonly used as a mirror of GnRH secretion (121, 123). However, pulsatile GnRH/LH release requires serial sampling and even LH measurements are hardly achievable with mouse blood volume (124). At a cellular level, the first challenge is anatomical: preserving the connectivity with relevant inputs (125), GnRH cell morphology (126), and tracking a neuron within the complexity of the ME (20). The second challenge is technical as methods for detection of quantal secretion are not applicable to GnRH neurons: (1) synaptically coupled neurons are recorded simultaneously in brain slices [reviewed in Ref. (127)], but GnRH neurons lack downstream partners, (2) patch clamp measurement of capacitive current is limited to soma and isolated nerve terminals (128, 129), therefore does not reflect hypophysiotropic GnRH secretion, and (3) fast-scan cyclic voltammetry (FSCV) is restricted to electrochemically active small neurotransmitters [reviewed in Ref. (130)].

Since techniques directly monitoring secretion cannot be applied to GnRH neurons, the alternative is to rely on the relationship between electrical activity, voltage-gated calcium channels, calcium, and secretion [reviewed in Ref. (131)] and use electrophysiology and calcium imaging of the GnRH cell bodies to assess GnRH secretion indirectly. The hypothesis of pulsatile secretion being intrinsic to GnRH neurons led to studies of electrical properties in GnRH neurons {GT1 cells (73); nasal explants (71); *ex vivo* GnRH neurons [reviewed in Ref. (40, 132, 133)]}. Although most GnRH neurons display autonomous firing of APs, firing is heterogeneous among GnRH neurons (126), far from an oscillatory activity that could trigger pulses every ~20 min (124). Even *in vivo* GnRH neurons exhibit heterogeneous behavior (114). The search for changes in the firing pattern *ex vivo*, i.e., increases in firing rate occurring at the same frequency as GnRH pulses, is rather inconclusive (134–136). Notably, in addition to intrinsic properties, it is assumed that each GnRH neuron contributes to consecutive GnRH pulses. Although experimentally activated GnRH neurons can trigger multiple LH pulses (88), this assumption has yet to be proven.

**Figure 1:**
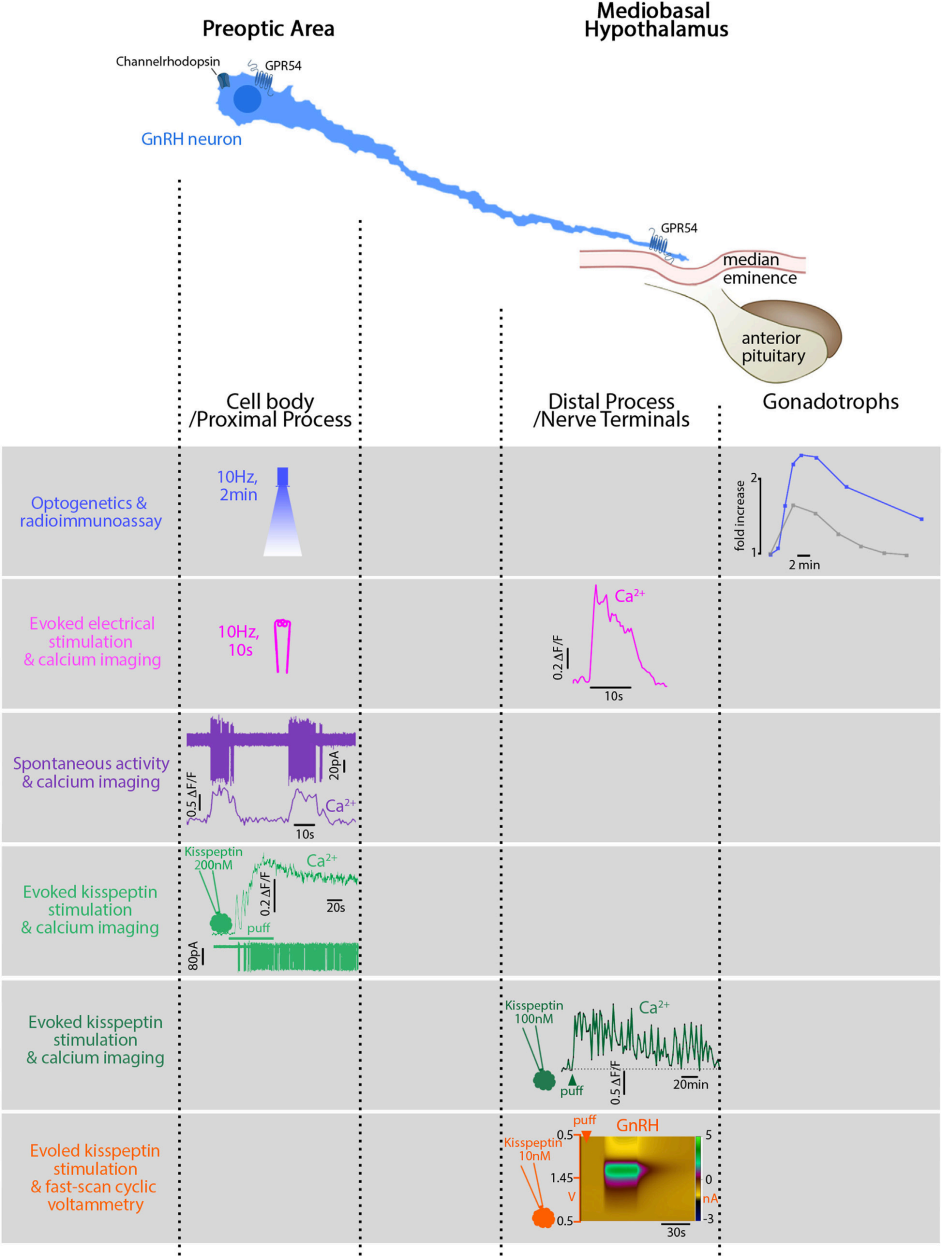
Relationship between electrical activity, intracellular calcium, and secretion in gonadotropin-releasing hormone (GnRH) neurons in mice. GnRH neurons can be divided into two main functional segments: cell body/proximal process, mainly in the preoptic area (POA), and distal process/nerve terminals, in the mediobasal hypothalamus. First row: blue light, flashed in the POA, electrically activates GnRH neurons expressing channelrhodopsin *in vivo*. The functional read-out reflecting GnRH secretion is the luteinizing hormone (LH) increase (blue trace) in the systemic circulation evoked by the gonadotrophs. The gray trace shows a spontaneously occurring LH pulse, much smaller. Second row: electrical stimulation of GnRH fibers activates GnRH neurons expressing genetically encoded calcium sensor GCaMP3 *in vitro* and evokes a calcium rise in the nerve terminals. Note the stimulus-restricted calcium increase. Third row: spontaneous action currents at the cell body evoke simultaneous rises in [Ca^2+^]_i_. Note: the difference between the frequency of spontaneous events (10 s, every 30 s) and the frequency of spontaneously occurring LH pulses [every 21 min in ovariectomized mice (124)]. Fourth row: Kisspeptin, locally applied at the cell body, binds to its cognate receptor, GPR54, and evokes a long-lasting calcium rise and train of action potentials (APs). Fifth row: Kisspeptin, locally applied at the nerve terminals, evokes a long-lasting calcium rise (>60 min) in GnRH neurons expressing genetically encoded calcium sensor GCaMP6s but no APs are required. Sixth row: Kisspeptin, locally applied at the nerve terminals, evokes secretion (~1 min). Figures adapted with permission of the authors [row 1 (88); row 2–5 (115); row 6 (141)].

Simultaneous recording from multiple GnRH neurons bypasses this assumption and shows *in vitro* relationships between synchronized [Ca^2+^]_i_ oscillations and GnRH pulses (70) or frequency of [Ca^2+^]_i_ oscillations and GnRH secretion (137). Recently, optogenetic activation of GnRH neurons defined the firing of GnRH neurons triggering LH secretion *in vivo* (88) (Figure 1). However, the predicament to linking an electrical event to a secretion, at a single-cell level, is the resolution for the detection of GnRH release. Calcium dynamics in GT1 cells correlate with FM1-43 uptake, i.e., secretion (138), but this observation cannot be extrapolated to native GnRH neurons with complex morphology and where GnRH release occurs from cell bodies and fibers (139, 140).

Modified FSCV, applicable to GnRH, is a step forward, providing secretion data from one to few GnRH neurons (140). It supports, at a smaller scale, the relationship between APs and secretion: increased firing rate evoked by hormonal status, recorded at the cell body (142), correlates with increased secretion, at the ME (140), highlighting the regulation of firing activity. Most importantly, it allows subcellular measurements and shows a site-specific regulation of GnRH release (141). Different regulation of somatodendritic and nerve terminal release is known in magnocellular neurons (143), but a new insight in GnRH neurons. In the POA (at bundles of proximal processes), increases in [Ca^2+^]_i_ evoked by sarco/endoplasmic reticulum calcium-ATPase blocker evoke GnRH release. While in the ME (at nerve terminals), APs must accompany such increases to evoke GnRH release (141). In addition, locally applied inositol triphosphate receptor blocker prevents kisspeptin-evoked GnRH release in the ME but not in the POA. In contrast, locally applied calcium channel blocker prevents kisspeptin-evoked GnRH release in the POA but not in the ME, where calcium and sodium channel blockers are necessary (141).

Subcellular electrophysiology and calcium imaging identify different functions at different locations in GnRH neurons (20, 115, 144) (Figure 1). APs initiate in the proximal process (144) and patterning occurs at the cell soma (145). APs propagate along the process (144) and elicit temporally restricted calcium rises at the nerve terminals (115). The activation of GnRH neurons in the POA triggering a GnRH/LH pulse *in vivo* illustrates this phenomenon (88). However, the straightforwardness stops with electrical stimuli. GnRH neurons become versatile when exposed to ligands. GnRH projections exhibit unique properties allowing local depolarizations to reshape APs along the way to the ME (20). Applied at the cell body, kisspeptin evokes a calcium rise, accompanied by APs (115). Although the calcium rise at the cell body is independent of firing (48, 115, 141), APs will travel and evoke a spike-dependent calcium rise at the nerve terminals (115). Applied at the nerve terminals, kisspeptin evokes a local calcium rise, independent of APs (115), and triggers GnRH secretion, even when sodium channel blockers are present (141).

**Figure 2:**
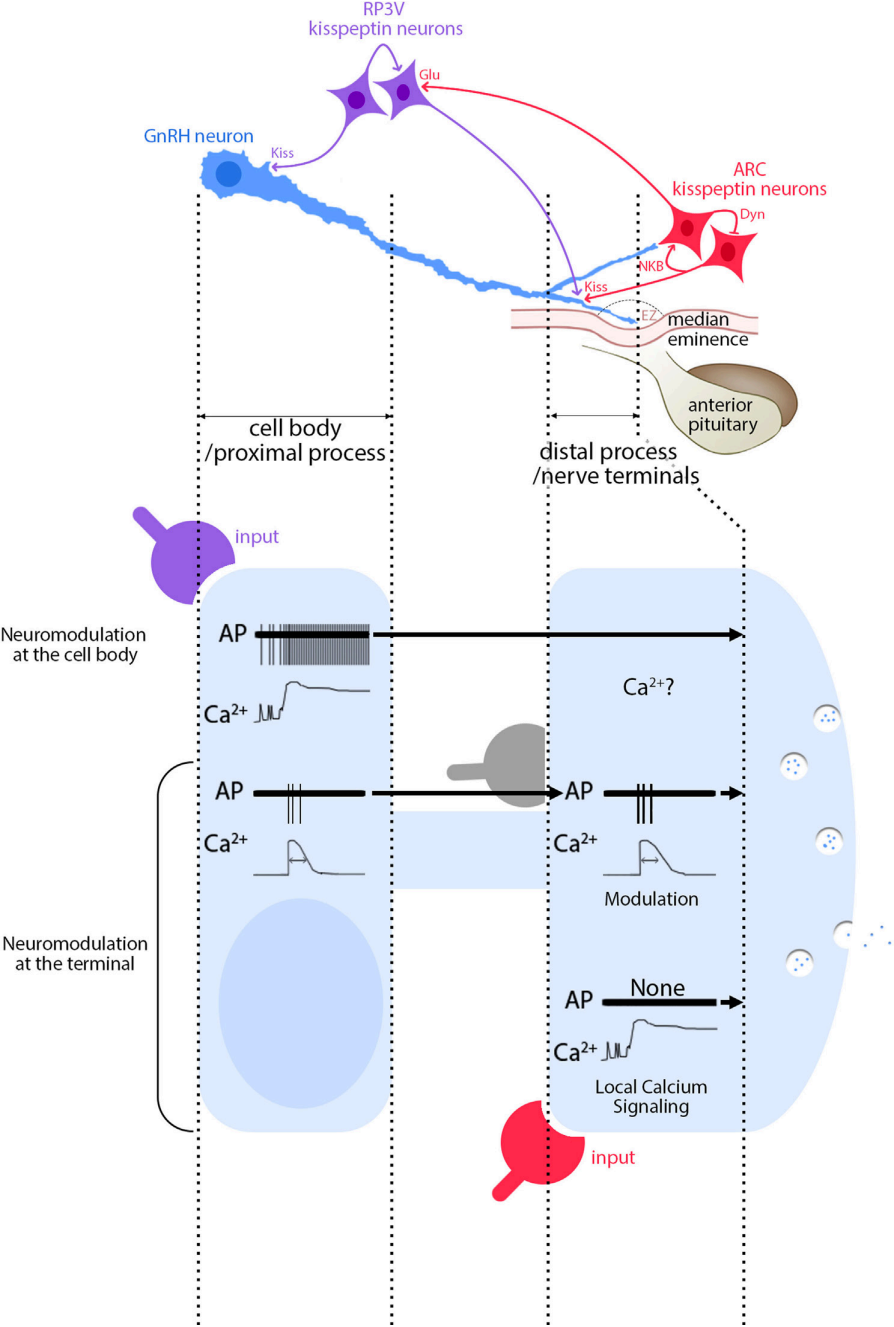
Functional consequences of segment-specific signaling in gonadotropin-releasing hormone (GnRH) neurons. Kisspeptin/GnRH signaling: GnRH neurons (blue) receive inputs from RP3V kisspeptin neurons (purple) at the cell body and at the distal process, outside the external zone (EZ) of the median eminence. In contrast, inputs from ARC kisspeptin neurons (red) are seen only at the distal process. Inputs from ARC to RP3V kisspeptin neurons are glutamatergic. Activity of ARC kisspeptin neurons relies upon an autoregulatory loop involving neurokinin B and dynorphin A. High magnification view of events: signals evoked at GnRH soma from RP3V kisspeptin neurons (purple) produce a long-lasting calcium rise and a train of action potentials (APs) that travel toward the nerve terminals (top traces). Based on Figure 1 (second row), an AP-dependent calcium rise would be expected, unless the calcium rise *via* GPR54 is autoregenerative, travels along the process and therefore would be AP independent. In contrast, signaling evoked at GnRH nerve terminals from ARC kisspeptin neurons (red) produces a long-lasting calcium rise, without APs (bottom traces). In addition, APs, accompanied by AP-dependent calcium rises, travel toward the nerve terminals and modulatory inputs such as glutamate (gray) can reshape the APs and possibly the concomitant calcium rises (middle traces). The traces are schematic and do not have scale bars.

Until today, the conundrum was with kisspeptin producing both a massive surge and timely restricted pulses. However, subcellular regulation in GnRH neurons provides new hypotheses for GnRH secretion (Figure 2). RP3V kisspeptin neurons innervating the GnRH cell body (118) probably initiate different response than ARC kisspeptin neurons innervating the nerve terminals (118), thus regulating GnRH secretion differently. For example, kisspeptin evokes a long-lasting calcium rise in nerve terminals (>60 min) (115) but FSCV detects GnRH release for ~1 min (141). Possibly, non-secreting calcium-dependent vesicle dynamics might follow calcium-evoked GnRH secretion at the nerve terminals (115, 129, 146). FSCV indicates kisspeptin-evoked secretion at the ME is specifically regulated and an increase in [Ca^2+^]_i_ is not the only requirement (141). Exocytosis involves many protein–protein interactions regulated by second messengers and phosphorylation (147). Kisspeptin triggers a complex signaling pathway (48, 148) that might allow it to define the relationship between calcium and secretion at GnRH nerve terminals.

## Conclusion

Our knowledge of the physiology of GnRH neurons is ever evolving and we should remain as naïve as possible when studying them. As in many other fields, the knowledge is limited by techniques and none of the “classical” tools available in neuroscience are readily usable for GnRH neurons. Even nowadays, the knowledge of GnRH neurons still suffers from the anatomical intricacy of the system. However, with creativity and tenacity, knowledge about GnRH neurons builds up and common assumptions fall: the simple bipolar GnRH neuron displays arborized distal processes, the scattered cell bodies are reunited with entwined dendrites, GnRH is released at the cell body, the processes become dendrons with merged features of axon and dendrite, and neuronal inputs relocate into differentially regulated GnRH neuron segments. I am positive the list will continue to grow as we try and understand the mechanism(s) underlying pulsatile GnRH secretion.

What do we need to unravel the mystery behind GnRH secretion? I believe the next step is to tailor genetic tools to target genetically encoded sensors such as GCaMP6s and pHuji, to GnRH neurons for simultaneous imaging of calcium dynamics and secretory vesicle fusion. This should allow for the deciphering of their precise relationship and the investigation of how intracellular signaling pathways downstream of GPCRs and other receptors can modulate this relationship.

## Author Contributions

The author confirms being the sole contributor of this work approved it for publication.

## Conflict of Interest Statement

The author declares that the research was conducted in the absence of any commercial or financial relationships that could be construed as a potential conflict of interest.
